# Does Coxsackievirus B3 Require Autophagosome Formation for Replication? Evidence for an Autophagosome-Independent Mechanism: Insights into Its Limited Potential as a Therapeutic Target

**DOI:** 10.3390/ph18121880

**Published:** 2025-12-11

**Authors:** Yun Ji Ga, Jung-Yong Yeh

**Affiliations:** 1 Department of Molecular Biomedical Science, Division of Life Sciences, College of Life Sciences and Bioengineering, Incheon National University, Harmony-ro 265, Yeonsu-gu, Incheon 22014, Republic of Korea; dkfmal92@inu.ac.kr; 2 Center for Brain-Machine Interface, Incheon National University, BioComplex, Harmony-ro 265, Yeonsu-gu, Incheon 22014, Republic of Korea; 3 Convergence Research Center for Insect Vectors, Incheon National University, Harmony-ro 265, Yeonsu-gu, Incheon 22014, Republic of Korea; 4 KU Center for Animal Blood Medical Science, College of Veterinary Medicine, Konkuk University, 120 Neungdong-ro, Gwangjin-gu, Seoul 05029, Republic of Korea

**Keywords:** antiviral, autophagosome, autophagy, coxsackievirus, virus

## Abstract

**Background/Objectives:** Coxsackievirus B3 (CVB3), a neurotropic enterovirus, is a major causative agent of viral encephalitis and myocarditis, yet no protective vaccine or effective antiviral therapy is currently available. Autophagy plays a dual role in viral infections, acting as both an antiviral defense and a process that can be exploited by certain viruses. Although CVB3 has been proposed to utilize autophagosomes as replication platforms, the underlying mechanisms remain controversial. **Methods:** In this study, we investigated the relationship between CVB3 replication and autophagosome formation under starvation-induced conditions and in ATG5 knockout cells. **Results:** While nutrient deprivation robustly induced autophagy, CVB3 infection did not trigger autophagosome formation. Moreover, viral replication proceeded efficiently in ATG5-deficient cells lacking autophagosomes. Pharmacological modulation of autophagy using rapamycin, a potent autophagy inducer, did not alter intracellular viral titers or protein expression, although extracellular viral release was modestly reduced. These results indicate that CVB3 replication occurs independently of autophagosome formation, suggesting that pharmacological targeting of autophagy provides limited therapeutic benefit. **Conclusions:** This study refines our understanding of autophagy as an antiviral target and highlights the need to identify alternative host-directed pathways for antiviral drug development.

## 1. Introduction

Coxsackievirus B3 (CVB3), a member of the genus *Enterovirus* within the family *Picornaviridae*, is a non-enveloped, positive-sense RNA virus [1,2]. CVB3 infection generally causes mild febrile or respiratory illness; however, neuroinvasive infection can occur, leading to central nervous system manifestations such as aseptic meningitis or viral encephalitis, as well as extra-neural complications including chronic myocarditis or cardiac failure [3,4]. Reports of CVB3-associated inflammation in the central nervous system, encompassing both meningitis and encephalitis, have been documented worldwide [5,6,7,8]. Despite its clinical relevance, no protective vaccine or effective antiviral therapy is currently available, posing a significant public health concern [9].

Autophagy is a well-conserved intracellular homeostatic process responsible for removing and recycling damaged cellular organelles and proteins [10,11,12]. Under normal conditions, autophagy occurs at a basal level, but it can be activated in response to stressors such as nutrient or energy deprivation. This process degrades cytoplasmic material into metabolites that can be reused for biosynthesis or energy production, thereby supporting cell survival [13]. Autophagy was considered a simple cellular mechanism for clearing dysfunctional proteins and organelles, but it is now recognized as a key player in diverse physiological and pathological contexts. In most tissues, autophagy is essential for continuous metabolic turnover, particularly under stressful conditions that threaten survival, such as nutrient scarcity or pathogen invasion [14,15,16]. Autophagy, as part of the host immune response, targets viral particles or components for degradation, thereby limiting viral replication [17]. However, many viruses, as intracellular parasites, can subvert or evade these antiviral responses. Increasing evidence indicates that autophagy can exhibit both antiviral and pro-viral roles in the replication of various viruses [18,19].

The dual role of autophagy in viral infection has prompted interest in developing antivirals that target this pathway. Therefore, selective modulation of autophagy has emerged as a promising strategy for antiviral therapy [20]. Nevertheless, non-specific induction of autophagy may lead to unintended consequences, including facilitating viral persistence. In this context, several studies have examined the interaction between CVB3 infection and host cell autophagy both in vitro and in vivo. Some reports have shown activation of the autophagy pathway, including autophagosome formation, following CVB3 infection [21,22,23], whereas others have presented conflicting results [24]. However, the specific autophagy pathways involved remain unclear. Understanding the complex interplay between viruses and host autophagy is crucial for the development of novel antiviral strategies. In this study, we sought to elucidate alterations in autophagy-related pathways, with particular emphasis on autophagosome formation, during CVB3 infection. Furthermore, we aimed to assess whether modulation of autophagosome formation could constitute a potential therapeutic target, thereby providing insights that may inform the development of novel strategies for the treatment of CVB3-associated diseases.

## 2. Results

### 2.1. CVB3 Infection Does Not Induce Autophagosome Formation

**Figure 1:**
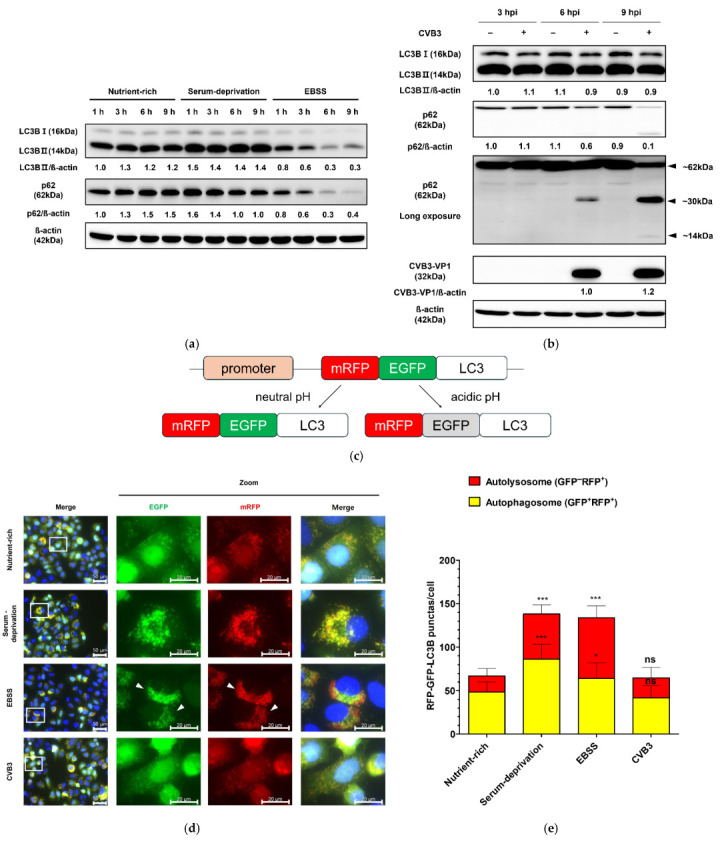

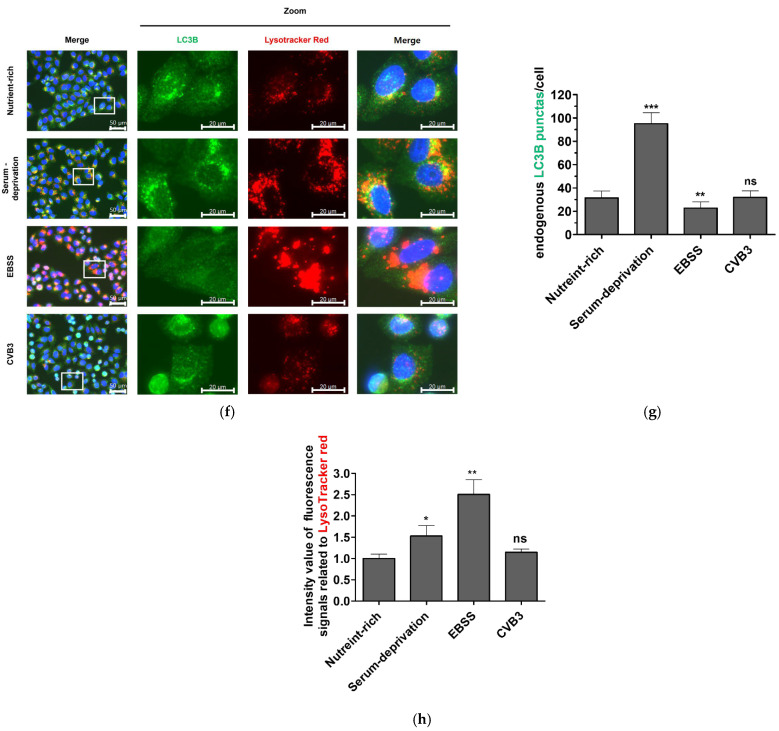
(**a**) Monitoring of autophagosome formation in starvation-induced and coxsackievirus B3 (CVB3)-infected HeLa cells at a multiplicity of infection of 0.1 by western blot. The expression levels of LC3B (LC3B-I and LC3B-II) and SQSTM1/p62 (p62) were analyzed by western blot at the indicated time points (h) under nutrient-rich and serum-deprived conditions. Serum deprivation was performed using DMEM medium identical to the nutrient-rich condition but lacking fetal bovine serum. EBSS medium is Earle’s balanced salt solution deficient in both serum and amino acids. (**b**) The expression levels of LC3B (LC3B-I and LC3B-II), p62, and CVB3 VP1 were assessed by western blot at the indicated hours post infection (hpi) in CVB3-infected or mock-infected cells. β-actin was used as a loading control. Relative protein levels were quantified using ImageJ software version 1.53 (NIH, Bethesda, MD, USA) and expressed as the ratio of target protein to β-actin. Black arrows indicate cleaved p62 fragments, and approximate molecular sizes are indicated. (**c**) Assessment of autophagosome formation in HeLa cells stably expressing mRFP-EGFP-LC3B. HeLa cells stably expressing mRFP-EGFP-LC3B were subjected to serum deprivation or infected with CVB3 for 9 h. Schematic representation of the mRFP-EGFP-LC3B reporter construct. (**d**) Representative immunofluorescence images showing exogenous mRFP-EGFP-LC3B localization. Nuclei were counterstained with Hoechst 33342 (blue). The white rectangle indicates the region magnified in the right panel. White arrows indicate attenuated GFP signal, which means autophagic flux. Scale bars: 50 μm (original image) and 20 μm (zoomed image). (**e**) Quantification of yellow puncta (GFP^+^/RFP^+^) and red puncta (GFP^−^/RFP^+^) per cell. (**f**) Representative immunofluorescence image of endogenous LC3B and Lysotracker red staining. Nuclei were counterstained with Hoechst 33342 (blue). The white rectangle indicates the region magnified in the right panel Scale bars: 50 μm (original image) and 20 μm (zoomed image). (**g**) Quantification of LC3B green puncta per cell. (**h**) Quantification of relative intensity value of fluorescence signals related to Lysotracker red. Data represent means ± SEM of three independent experiments, with 10 cells analyzed per assay. Statistical significance was determined using a *t*-test (* *p* < 0.05; ** *p* < 0.01; *** *p* < 0.001; ns, not significant).

To monitor autophagosome formation in HeLa cells, we generated a HeLa cell line stably expressing monomeric red fluorescent protein(mRFP)–enhanced green fluorescent protein (EGFP)–LC3B. HeLa cells induced to autophagy by serum deprivation showed an increase in LC3BII and a slight decrease in p62 protein compared to cells in a nutrient-rich medium, whereas cells in serum- and amino acid-deprived EBSS medium showed a significant decrease in both LC3BII and p62 protein over time, indicating complete autophagic flux (Figure 1a). In contrast, CVB3-infected cells showed no change in LC3B-II levels relative to mock-infected controls (Figure 1b). At 9 h, serum starvation and EBSS induced a marked increase in both yellow puncta (autophagosomes, GFP^+^/RFP^+^) and red puncta (autolysosomes, GFP^−^/RFP^+^) (Figure 1c), demonstrating that the mRFP-EGFP-LC3B stably expressing HeLa cell line appropriately responded to autophagy induction. These findings were consistent with the western blot results showing elevated LC3B-II and reduced p62 levels under starvation conditions (Figure 1a). In contrast, LC3B-II levels remained unchanged under nutrient-rich conditions in CVB3-infected cells, which was in agreement with the mRFP-EGFP-LC3B reporter assay showing no increase in puncta formation (Figure 1b,d,e). Furthermore, serum deprivation and EBSS medium increased Lysotracker red fluorescence, whereas CVB3-infected cells showed similar LC3B distribution and Lysotracker red staining to nutrient-rich controls (Figure 1f,g,h). Together, these results demonstrate that CVB3 infection does not induce autophagosome formation beyond the basal level. Collectively, these findings indicate that CVB3 infection does not enhance autophagosome formation compared with the nutrient-rich basal state.

### 2.2. CVB3 Does Not Affect the Upstream Akt/mTOR/p70S6K Signaling Pathway

**Figure 2:**
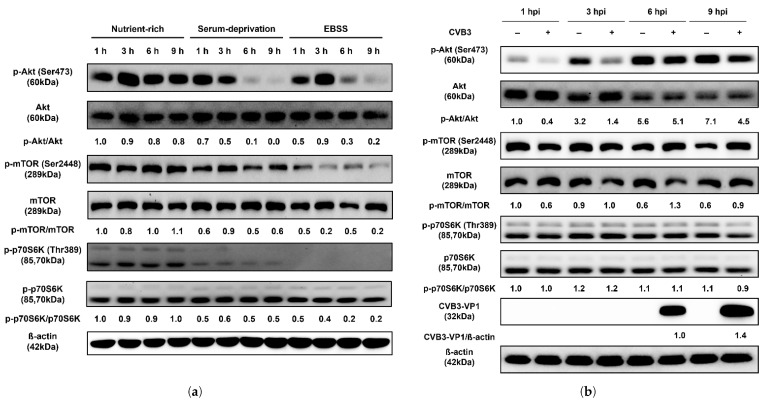

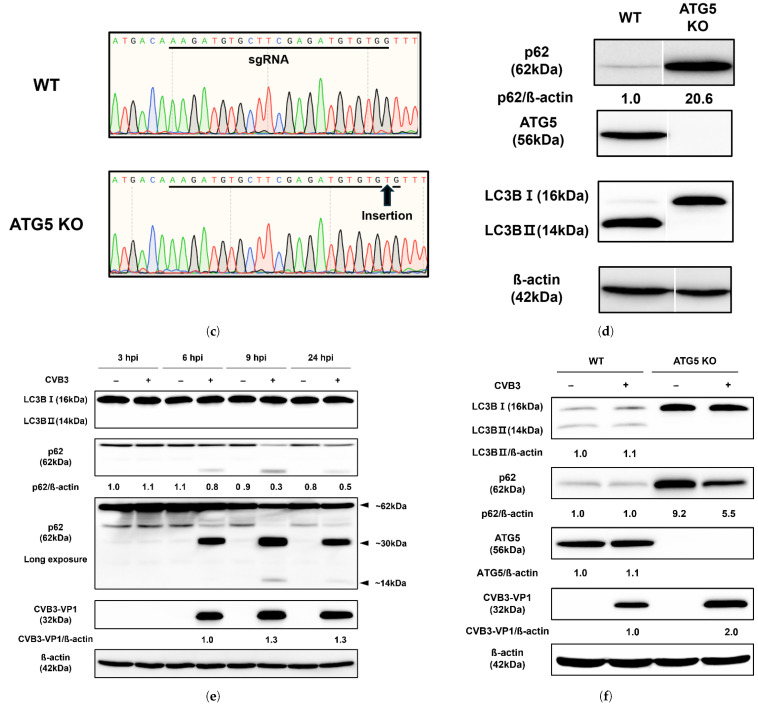
(**a**) CVB3 does not affect the upstream Akt/mTOR/p70S6K signaling pathway. Western blot analysis of the Akt/mTOR/p70S6K signaling pathway in HeLa cells subjected to serum deprivation. The expression levels of p-Akt, Akt, p-mTOR, mTOR, p-p70S6K, and p70S6K were examined at the indicated hours (h) under nutrient-rich and two starvation conditions (serum-deprivation and EBSS). Serum-deprivation uses the same DMEM medium as nutrient-rich, but without fetal bovine serum. EBSS medium is Earle’s balanced salt solution deficient in both serum and amino acids. CVB3-VP1 proteins were detected by western blot after indicated hours post infection (hpi) with or without CVB3 at a multiplicity of infection of 0.1. β-actin was used as an internal control. Relative protein levels were quantified using ImageJ software (NIH, Bethesda, MD, USA) and expressed as the ratio of phosphorylated to total protein. (**b**) Western blot analysis of the Akt/mTOR/p70S6K signaling pathway in HeLa cells infected with CVB3. Protein levels (p-Akt, Akt, p-mTOR, mTOR, p-p70S6K, and p70S6K) were detected at the indicated hours post infection (hpi) in CVB3-infected and mock-infected cells. β-actin was used as an internal control. Relative protein levels were quantified using ImageJ and expressed as the ratio of phosphorylated to total protein and the ratio of CVB3-VP1 protein to β-actin. (**c**) Generation of ATG5 knockout (KO) HeLa cells. DNA sequencing confirmed a mutation in ATG5 KO cells, with the black arrow indicating the insertion of a nucleotide (T) within the sgRNA target site. (**d**) Equal amounts of lysates from wild-type (WT) and ATG5 KO HeLa cells were analyzed using antibodies against SQSTM1/p62 (p62), ATG5, and LC3B (LC3B-I and LC3B-II). β-actin served as an internal control. Image splicing was performed only between non-adjacent lanes without altering the order of samples, and straight cutting white lines were applied. (**e**) The expression levels of LC3B (LC3B-I and LC3B-II), SQSTM1/p62 (p62), and CVB3-VP1 were analyzed by western blot at 3–24 hpi with or without CVB3. (**f**) ATG5 knockout demonstrates that autophagosome formation is dispensable for CVB3 replication. The expression levels of LC3B, p62, ATG5, and CVB3-VP1 proteins were detected by western blot after 24 hpi with or without CVB3 in WT HeLa cells and ATG5 KO HeLa cells. Relative protein levels were quantified with ImageJ software (NIH, Bethesda, MD, USA) and expressed as the ratio of target protein to β-actin.

Since autophagosome formation was not observed in HeLa cells following CVB3 infection, unlike under nutrient deprivation, we next examined the phosphorylation status of the Akt/mTOR/p70S6K signaling pathway. Compared with nutrient-rich conditions, serum starvation reduced phosphorylation of mTOR, a key negative regulator of autophagy initiation, beginning at 6 h. And cells treated with EBSS medium, which showed more severe autophagy induction compared to serum starvation in previous experiments, rapidly decreased the phosphorylation of mTOR from 3 h after treatment (Figure 2a). Phosphorylation of Akt, an upstream regulator of mTOR, was also markedly decreased at 6 h, while phosphorylation of p70S6K, a downstream target of mTOR and negative regulator of autophagy, was drastically reduced as early as 1 h after starvation. In contrast, the total protein levels of Akt, mTOR, and p70S6K remained unchanged. These results indicate that nutrient deprivation rapidly inhibits phosphorylation of the Akt/mTOR/p70S6K pathway and induces autophagy within 9 h. By contrast, CVB3-infected HeLa cells showed no reduction in phosphorylation of Akt, mTOR, or p70S6K, even after 6 h when VP1 protein became detectable, relative to mock-infected controls, indicating that this signaling profile diverges from the inhibitory phosphorylation pattern observed under starvation (Figure 2b). These results are consistent with the findings of the mRFP-EGFP-LC3B reporter assay described above, which demonstrated the absence of autophagosome formation during CVB3 infection.

### 2.3. ATG5 Knockout Reveals Autophagosome Formation Is Dispensable for CVB3 Replication

Based on our finding that CVB3 infection does not induce autophagosome formation, we generated an ATG5 knockout (KO) HeLa cell line using the CRISPR/Cas9 system to investigate the role of autophagosomes in CVB3 replication. Genome sequencing of the ATG5 KO cells revealed the insertion of a single thymine nucleotide within the sgRNA target site compared with wild-type (WT) HeLa cells (Figure 2c). Western blot analysis confirmed that this mutation abolished ATG5 protein expression in ATG5 KO cells (Figure 2d). No conversion of LC3B-I to LC3B-II was detected in ATG5 KO cells, indicating that autophagosome formation was blocked in the absence of ATG5. In CVB3-infected ATG5 KO cells, VP1 protein expression was detected at 6 h post-infection, similar to WT cells (Figure 1b and Figure 2e). In addition, the p62 protein decreased over time in CVB3-infected cells compared to un-infected cells, and fragments of approximately 30 kDa and 14 kDa were also identified (Figure 2e). After 24 h, ATG5 KO cells lacking ATG5 protein and LC3B-II nonetheless exhibited higher levels of VP1 compared with WT cells (Figure 2f). These findings demonstrate that autophagosome formation is not required for CVB3 infection and may even restrict viral replication.

### 2.4. Effect of Rapamycin-Induced Autophagy on CVB3 Replication

To evaluate the effect of rapamycin, an mTOR inhibitor and potent autophagy inducer, on CVB3 replication, we first determined a non-toxic concentration using a soluble tetrazolium salt assay. HeLa cells were treated with various concentrations of rapamycin (25–1600 nM) for 24 h, which showed a dose-dependent reduction in cell viability compared with controls. Based on these results, 200 nM rapamycin was selected, as this concentration maintained >90% cell viability after 24 h.

**Figure 3:**
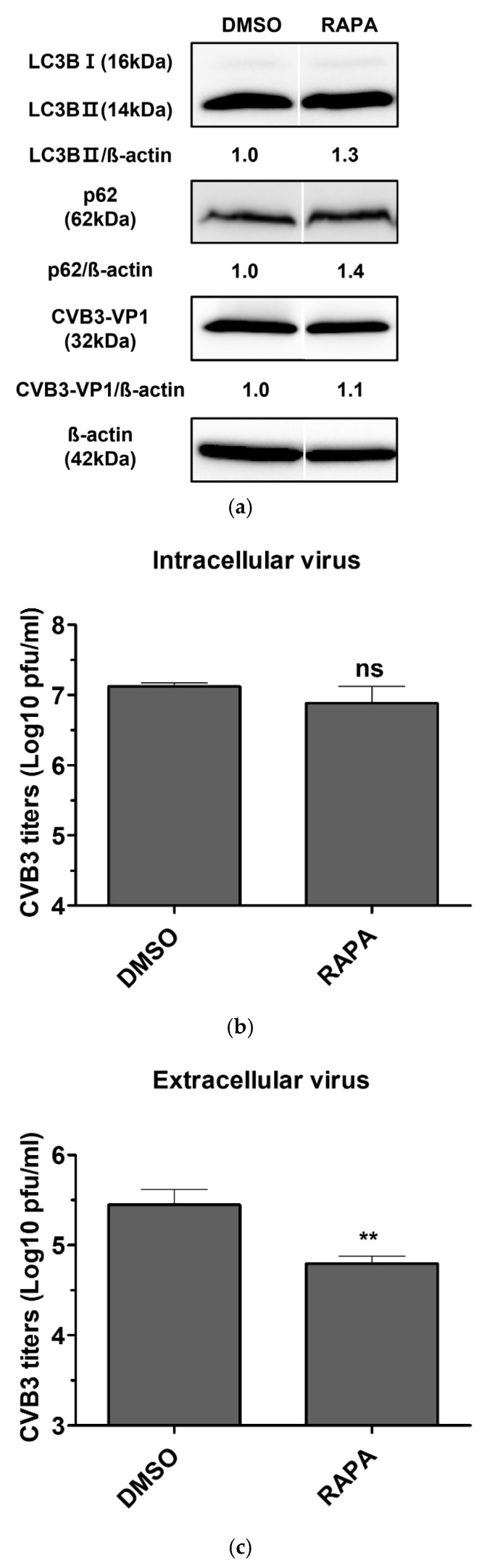
(**a**) Evaluation of the antiviral activity of rapamycin in coxsackievirus B3 (CVB3)-infected HeLa cells. HeLa cells were infected with CVB3 at a multiplicity of infection of 0.1 and treated with 200 nM rapamycin (RAPA) for 24 h. Cells treated with 0.1% DMSO alone served as vehicle controls. Cell lysates were analyzed by western blot using antibodies against SQSTM1/p62 (p62), LC3B (LC3B-I and LC3B-II), and CVB3-VP1. β-actin was used as an internal control. Relative protein levels were quantified using ImageJ software (NIH, Bethesda, MD, USA) and expressed as the ratio of target protein to β-actin. Image splicing was performed only between non-adjacent lanes without altering the order of samples, and straight cutting white lines were applied. (**b**) Intracellular viral titers were determined by plaque assay from cell lysates. (**c**) Extracellular viral titers were determined by plaque assay from culture supernatants. Viral titers were expressed as plaque-forming units (PFU) per milliliter (ml) and presented as log10 values, quantified in Vero cells. Data represent the mean ± SD of three independent experiments. Statistical significance was determined by *t*-test (**, *p* < 0.01; ns, not significant).

HeLa cells infected with CVB3 were then treated with 200 nM rapamycin for 24 h. Viral titers were quantified separately in extracellular virus (supernatants) and intracellular virus (cell lysates). Rapamycin did not alter viral protein expression compared with the DMSO control, indicating that the antiviral effect of rapamycin-induced autophagy activation was limited (Figure 3a). Consistently, viral titration assays showed no significant change in intracellular virus levels (Figure 3b). In contrast, extracellular virus was reduced by approximately six-fold following rapamycin treatment (Figure 3c), suggesting that rapamycin impairs viral release rather than intracellular replication, independently of autophagy induction.

## 3. Discussion

Autophagy is an evolutionarily conserved intracellular degradation pathway in eukaryotic cells, in which cytoplasmic components are sequestered by double-membrane vesicles (autophagosomes) and subsequently degraded following fusion with lysosomes [25]. This regulated process maintains cellular homeostasis under both normal and stressful conditions [11,26,27]. During viral infection, autophagy can serve as an innate defense mechanism to eliminate pathogens and promote cell survival [28]. However, many viruses have evolved strategies to evade or exploit autophagy to enhance their replication [29]. Although increasing evidence suggests a complex interplay between CVB3 and the host autophagy machinery, the precise role of autophagy in CVB3 infection remains controversial. In this study, we compared nutrient starvation, a classical inducer of autophagy, with CVB3 infection in HeLa cells. While serum deprivation robustly induced autophagosome formation, CVB3 infection did not increase autophagosome production beyond basal levels (Figure 1 and Figure 2). Contrary to the prevailing hypothesis that CVB3 infection promotes autophagosome formation to serve as a platform for viral RNA genome replication [23,30,31], we observed no increase in autophagosome production comparable to that induced by nutrient starvation.

Furthermore, we examined the Akt/mTOR/p70S6K signaling pathway, a key regulator of autophagy that is modulated in response to nutrient deprivation or viral infections such as coxsackievirus A16 and enterovirus 71 [32,33]. While several studies have reported that CVB3 infection in HeLa cells induces autophagy by inhibiting mTOR [34,35], other reports found no significant alteration in mTOR activity following infection [30]. In our study, nutrient starvation markedly reduced phosphorylation of Akt, mTOR, and p70S6K, consistent with induction of autophagy. In contrast, the minor fluctuations in phosphorylation detected during CVB3 infection did not exceed the variability typically observed under basal conditions, indicating no meaningful suppression of the pathway, which is consistent with the absence of autophagosome formation under the same conditions (Figure 2). Western blot densitometry was performed from a single biological experiment with technical replicates. Although the number of biological replicates is limited, the observed trends were consistent across independent assays, supporting the validity of the conclusions.

Autophagosome formation has been proposed as a common feature of several RNA virus infections (e.g., CVB3, poliovirus, coxsackievirus B4, coxsackievirus A16, enterovirus 71, and enterovirus D68), where it is thought to facilitate viral replication by providing a membranous platform for RNA genome synthesis [23,30,31]. In contrast, our results demonstrate that CVB3 infection does not induce autophagosome formation via the classical nutrient deprivation–induced pathway. Moreover, CVB3 replication was not impaired in ATG5 KO HeLa cells, in which autophagosome formation is restricted, confirming that viral infection proceeds independently of autophagy (Figure 2). These findings are partially consistent with the report by Alirezaie et al., which suggested that the bilayer structures observed during CVB3 infection originate from multiple sources, with autophagic membranes representing only one possible contributor [36]. Consistent with this, we found that the p62 protein, a marker of autophagic flux and a ubiquitinated protein that targets autophagosomes for degradation, was cleaved by viral infection in a manner independent of autophagy (Figure 1b and Figure 2e). These results are consistent with previous findings that p62 can be cleaved by the protease 2Apro of CVB3 Kandolf strain [37]. Selective autophagy receptors, such as p62, play a key role in cellular defense strategies against invading pathogens such as viruses, as they have been demonstrated to be able to target invaders for lysosomal degradation as part of the innate immune response [26,38]. Accordingly, our results support previous in vivo evidence showing that ATG5 depletion does not prevent CVB3 replication [22].

Based on our findings that CVB3 infection does not induce classical autophagy, such as that triggered by nutrient deprivation, and that viral replication proceeds independently of autophagosomes formed during early autophagy, we next examined the effects of autophagy induction on CVB3 infection using rapamycin, an mTOR inhibitor and potent autophagy inducer [39]. Rapamycin enhances autophagosome formation by suppressing mTOR activity, a negative regulator of autophagy, and has been reported in previous studies to promote the replication of polioviruses and foot-and-mouth disease virus, both of which benefit from autophagosome formation [40,41]. However, in this study, rapamycin treatment only modestly reduced infectious extracellular CVB3 titers, while intracellular virus levels and viral protein expression remained unchanged (Figure 3). These findings are consistent with our observation that CVB3 infection is independent of autophagosome production, suggesting that induction of autophagy and the resulting autophagosome formation do not provide a significant advantage for CVB3 replication. Collectively, our results demonstrate that increasing autophagosome formation is not an effective antiviral strategy against CVB3 infection. Nevertheless, because rapamycin primarily targets mTOR and can affect multiple cellular processes beyond autophagy, future studies will incorporate pharmacological modulators that selectively regulate different stages of the autophagy pathway. This approach will enable us to more precisely determine whether any specific phase of autophagy functionally influences CVB3 replication and viral release.

Although our HeLa cell–based system offers a robust model for assessing the necessity of autophagosome formation during early CVB3 infection, the applicability of these findings to other physiologically relevant cell types remains uncertain. To address this limitation, future studies will examine additional CVB3-permissive models, including cardiomyocytes and neuronal cell lines, to determine whether the autophagy-independent phenotype observed in HeLa cells is conserved across diverse host environments. Furthermore, because CVB3 strains differ in virulence and tissue tropism, we plan to evaluate multiple viral isolates in subsequent experiments to assess whether this phenotype is maintained across distinct CVB3 genetic backgrounds.

In conclusion, our findings establish that CVB3 infection in host cells proceeds independently of autophagosome formation and does not benefit from classical autophagy-inducing signals. Although previous some studies have suggested that autophagosomes may serve as replication platforms for CVB3, our findings underscore that modulation of autophagosome formation is not a viable antiviral approach. These insights may guide the development of more precise therapeutic strategies for CVB3-associated diseases.

## 4. Materials and Methods

### 4.1. Cell Culture and Drug Treatment

HeLa CCL-2™ cells were obtained from the American Type Culture Collection (ATCC, Rockville, MD, USA) and maintained in Dulbecco’s Modified Eagle’s Medium (DMEM, Invitrogen Life Technologies, Carlsbad, CA, USA) supplemented with 10% fetal bovine serum (FBS, Merck Millipore, Darmstadt, Germany), 100 U/mL penicillin, and 100 μg/mL streptomycin at 37 °C in a humidified incubator with 5% CO_2_. Vero CCL-81™ cells (ATCC) were cultured in Minimum Essential Medium α without nucleosides (MEM-α, Invitrogen Life Technologies, Carlsbad, CA, USA) supplemented with 5% FBS, 100 U/mL penicillin, and 100 μg/mL streptomycin under the same conditions. Rapamycin(R8781) was dissolved in dimethyl sulfoxide (DMSO) to a final concentration of 0.1%. Cells were treated with indicated concentrations and for indicated time periods. As control, cells were treated with appropriate DMSO concentrations.

### 4.2. Virus

A CVB3 clinical isolate from South Korea in 2002, which was confirmed to belong to genogroup D [42], was provided by the National Pathogen Culture Centre (Osong-eup, Cheongju-si, Republic of Korea). For virus propagation, HeLa cells were grown to 80–85% confluence and infected with CVB3 at a multiplicity of infection (MOI) of 0.1 in serum-free medium. After 1 h of absorption, the cells were washed with PBS and replenished with fresh medium containing 10%FBS. Infected cells showing cytopathic effects were harvested and subjected to three cycles of freeze–thawing between –80 °C and 37 °C. The supernatant was clarified by centrifugation, aliquoted, and stored at –80 °C until use. Viral titers were determined by plaque assays on Vero cells.

### 4.3. Induction of Autophagy Activation (Autophagosome Formation)

To accurately assess autophagy induction by CVB3 infection, nutrient-rich medium in HeLa cells was replaced with starvation-inducing conditions. Specifically, Earl’s Balanced Salt Solution (EBSS), which is deficient in both amino acids and serum, and serum-deprived medium were used as positive controls for autophagy induction, both of which are known to trigger autophagy within a short time frame. The conversion of LC3B-II, a marker of autophagosome formation, and the degradation of p62, a marker of autophagic flux, were then examined over time following serum deprivation. In addition, rapamycin was used to induce autophagosome formation, a key step in the autophagy process [43,44]. Rapamycin was dissolved in dimethyl sulfoxide (DMSO) at a final concentration of 0.1%.

### 4.4. Western Blot Analysis

Western blot analysis was performed to examine changes in intracellular autophagy-related proteins in response to CVB3 infection. Cells were lysed on ice for 20 min using M-PER reagent (Invitrogen Life Technologies, Carlsbad, CA, USA) supplemented with a protease/phosphatase inhibitor cocktail (Roche Diagnostics, Indianapolis, IN, USA) on ice for 20 min. Lysates were collected by scraping and clarified by centrifugation at 14,000× *g* for 15 min at 4 °C. Protein concentrations were determined using the BCA Protein Assay Kit (Intron Biotechnology, Seongnam-si, Republic of Korea). Equal amounts of protein were resolved by sodium dodecyl sulfate-polyacrylamide gel electrophoresis (SDS-PAGE) and transferred to polyvinylidene difluoride membranes (Bio-Rad Laboratories, Hercules, CA, USA). Membranes were blocked for 1 h at room temperature with 5% non-fat milk in Tris-buffered saline containing 0.05% Tween-20 (TBST), and subsequently incubated overnight at 4 °C with primary antibodies, followed by a 1 h incubation with the corresponding secondary antibodies. Immunoreactive bands were visualized using enhanced chemiluminescence (GE Healthcare, Waukesha, WI, USA). Primary antibodies included anti-LC3B (Sigma-Aldrich, Saint Louis, MO, USA); anti-SQSTM1/p62, anti-β-actin (Santa Cruz Biotechnology, Dallas, TX, USA); anti-CVB3 VP1 (Mediagnost, Reutlingen, Germany); and anti-Akt, anti–p-Akt, anti-mTOR, anti–p-mTOR, anti-p70S6K, anti–p-p70S6K, anti-rabbit IgG, and anti-mouse IgG (Cell Signaling Technology, Danvers, MA, USA). All experimental samples used for each comparative analysis were run on the same blot. For clarity in presentation, any lanes that were removed are clearly marked with an X in the corresponding raw images. Lane splicing was performed only between non-adjacent lanes without altering the sample order, and straight white cutting lines were applied to indicate the boundaries of the spliced regions.

### 4.5. Fluorescent LC3 Reporter Assay for Autophagosome Visualization

When the cells reached 70–90% confluence, they were transfected with 1 μg of an mRFP-EGFP-LC3B–encoding plasmid (ptfLC3 #21074, Addgene, MA, USA) using Lipofectamine 3000 (Invitrogen Life Technologies, Carlsbad, CA, USA). The cells were cultured at 37 °C in a humidified incubator with 5% CO_2_ using serum-free Opti-MEM (Invitrogen Life Technologies, Carlsbad, CA, USA). After 2 days, 500 μg/mL of G418 was added for selection. Colonies with distinct borders appeared after 5 days, from which single colonies were isolated and seeded into 96-well plates. Clones were further cultured for 10 days in the presence of 500 μg/mL G418. Stable mRFP–EGFP–LC3B–expressing clones were examined for co-localization of red and green puncta using an epifluorescence microscope (Carl Zeiss, Jena, Germany) and analyzed with AxioVision software version 4.0 (Carl Zeiss). Assessment of autophagic flux by different short-term stimuli conditions in HeLa cells stably expressing exogenous mRFP-EGFP-LC3B. HeLa cells stably expressing mRFP-EGFP-LC3B treated with serum-deprivation, EBSS, or infected CVB3 with 0.1 MOI for 9 h. Autophagosomes and autolysosomes were distinguished as follows: yellow puncta (GFP^+^ and RFP^+^) indicated autophagosomes, whereas red puncta (GFP^–^ and RFP^+^) indicated autolysosomes. The number of puncta per cell was quantified using ImageJ software (NIH, Bethesda, MD, USA). Quantification was performed from 10 cells per group, and the same trend was consistently observed across all replicates.

### 4.6. Immunofluorescence

To visualize autolysosomes bound to acidified lysosomes, we performed Lysotracker red staining, which accumulates in acidic organelles, and co-stained with endogenous LC3B. HeLa cells were grown on chamber slide incubated with with serum-deprivation, EBSS, or with serum-deprivation, EBSS, or infected CVB3 with 0.1 MOI for 9 h. Subsequently, the cells were incubated with 500 nM Lysotracker red DND-99 at 37 °C for 1 h and washed with cold PBS and fixed in 4% paraformaldehyde for 10 min. The fixed cells were permeabilized with 0.1% Triton X-100 in in PBS with 0.05% Tween20 for 10 min, blocked with 1% BSA in PBST for 30 min at 25 °C, and incubated overnight at 4 °C with LC3B antibody (Cell Signaling Technology, Danvers, MA, USA). After washing, the cells were incubated with Goat Anti-Rabbit IgG H&L FITC (Abcam, Cambridge, MA, USA) for 1 h at 25 °C. The nuclei were stained with Hoechst 33342 for 10 min at 25 °C. The cells were visualized using an epifluorescence microscope (Carl Zeis) and analyzed using Axio Vision software version 4.0 (Carl Zeiss). ImageJ software (NIH) was used to count the intensity of Lysotracker red and the number of LC3B puncta in each cell. Quantification was performed from 10 cells per group, and the same trend was consistently observed across all replicates

### 4.7. Production of ATG5 KO Cell Lines

We employed the CRISPR/Cas9 system to generate ATG5 knockout (KO) HeLa cell line. When the cells reached 70–90% confluence, they were co-transfected with 1 μg each of an ATG5 sgRNA plasmid (sgRNA sequence: AAGATGTGCTTCGAGATGTGTGG), pRGEN-Cas9-CMV, and pHRS_HumanATG5_CMV using Lipofectamine 3000 (Invitrogen Life Technologies, Carlsbad, CA, USA). The cells were maintained at 37 °C in a humidified incubator with 5% CO_2_ in serum-free Opti-MEM (Invitrogen Life Technologies, Carlsbad, CA, USA). After 2 days, 500 μg/mL hygromycin was added for selection. Colonies with distinct borders appeared after 5 days, from which single-cell colonies were isolated, seeded into 96-well plates, and cultured for 10 days. Selected colonies were divided into two groups: one maintained in culture and the other analyzed by sequencing and western blotting to confirm the absence of ATG5 protein. Exon 2 of the ATG5 gene was amplified by PCR using the following primer set: 5′-TGTCAGGATTCACAGGGTATAG-3′ and 5′-GCCTCCAAGTTCTTACAGC-3′. Purified PCR products were submitted to Cosmogenetech Inc. (Seoul, Republic of Korea) for genomic sequencing.

### 4.8. Cell Viability Assay

Cell viability in response to autophagy-modulating drugs was assessed using the EZ-Cytox Cell Viability Assay Kit (Daeillab Service, Seoul, Republic of Korea), which is based on a water-soluble tetrazolium salt assay. Briefly, 100 μL of a cell suspension (2 × 10^5^ cells/well) was seeded into 96-well plates. After 24 h, cells were treated with rapamycin and incubated for an additional 24 h. EZ-Cytox solution (10 μL) was then added to each well and incubated for 1 h at 37 °C. Absorbance was measured at 450 nm using a microplate spectrophotometer (Spectra Max 190, Molecular Devices, Sunnyvale, CA, USA). Cell viability (%) was calculated as (As/Ac) × 100, where As is the absorbance of wells containing cells, culture medium, EZ-Cytox solution, and stimulants, and Ac is the absorbance of wells containing cells, culture medium, and EZ-Cytox solution.

### 4.9. Plaque Assay

Vero cells, a well-established model for plaque assay quantification of enteroviruses, were used to determine CVB3 titers following treatment with autophagy-modulating drugs. Supernatants and lysates from CVB3-infected cells were serially diluted 10-fold and inoculated onto 90–95% confluent monolayers of Vero cells. After 1 h adsorption, the cells were washed with PBS and overlaid with 2× DMEM (Welgene, Gyeongsan, Republic of Korea) containing 2% agar (Lonza, Walkersville, MD, USA). Cultures were incubated at 37 °C for 72 h, fixed with 3.75% formaldehyde (Sigma-Aldrich), and stained with 1% crystal violet (Lugen Sci, Seoul, Republic of Korea). Plaques were counted, and viral titers were calculated as plaque-forming units (PFU) per milliliter. To minimize error, dilutions yielding 10–100 plaques were used for quantification, and results were expressed as log10 PFU/mL. Extracellular titers (supernatants) and intracellular titers (cell lysates) were determined in triplicate.

### 4.10. Statistical Analysis

Three independent experiments were performed in triplicate, and statistical analyses were conducted using GraphPad Prism 9.0.0 (GraphPad Software, San Diego, CA, USA). Statistical significance was evaluated using Student’s *t*-test, one-way ANOVA with Bonferroni’s multiple comparison test, or two-way ANOVA with Bonferroni’s post hoc test, as appropriate. A *p* value of <0.05 was considered statistically significant.

## Data Availability

The original contributions presented in this study are included in the article. Further inquiries can be directed to the corresponding author.

## Abbreviations

CVB3	coxsackievirus B3
GFP	green fluorescent protein
KO	knockout
RFP	red fluorescent protein
WT	wild-type

## References

[1] Lindberg A.M., Stålhandske P.O., Pettersson U. (1987). Genome of coxsackievirus B3. Virology.

[2] Tapparel C., Siegrist F., Petty T.J., Kaiser L. (2013). Picornavirus and enterovirus diversity with associated human diseases. Infect. Genet. Evol..

[3] Cooper L.T. (2009). Myocarditis. N. Engl. J. Med..

[4] Sin J., Mangale V., Thienphrapa W., Gottlieb R.A., Feuer R. (2015). Recent progress in understanding coxsackievirus replication, dissemination, and pathogenesis. Virology.

[5] Chu P.Y., Ke G.M., Chen Y.S., Lu P.L., Chen H.L., Lee M.S., Chen B.C., Huang T.S., Li Y.C., Chou L.C. (2010). Molecular epidemiology of Coxsackievirus B3. Infect. Genet. Evol..

[6] Lee C.J., Huang Y.C., Yang S., Tsao K.C., Chen C.J., Hsieh Y.C., Chiu C.H., Lin T.Y. (2014). Clinical features of coxsackievirus A4, B3 and B4 infections in children. PLoS ONE.

[7] Wong A.H., Lau C.S., Cheng P.K., Ng A.Y., Lim W.W. (2011). Coxsackievirus B3-associated aseptic meningitis: An emerging infection in Hong Kong. J. Med. Virol..

[8] Calderón K.I., Díaz-de Cerio M., Otero A., Muñoz-Almagro C., Rabella N., Martínez-Rienda I., Moreno-Docón A., Trallero G., Cabrerizo M. (2016). Molecular epidemiology of coxsackievirus B3 infection in Spain, 2004–2014. Arch. Virol..

[9] Mone K., Lasrado N., Sur M., Reddy J. (2023). Vaccines against Group B Coxsackieviruses and Their Importance. Vaccines.

[10] Levine B., Kroemer G. (2008). Autophagy in the pathogenesis of disease. Cell.

[11] Choi A.M., Ryter S.W., Levine B. (2013). Autophagy in human health and disease. N. Engl. J. Med..

[12] De Duve C. (1963). The lysosome. Sci. Am..

[13] Yorimitsu T., Klionsky D.J. (2005). Autophagy: Molecular machinery for self-eating. Cell Death Differ..

[14] Dooley H.C., Razi M., Polson H.E., Girardin S.E., Wilson M.I., Tooze S.A. (2014). WIPI2 links LC3 conjugation with PI3P, autophagosome formation, and pathogen clearance by recruiting Atg12-5-16L1. Mol. Cell.

[15] Kim K.H., Lee M.S. (2014). Autophagy—A key player in cellular and body metabolism. Nat. Rev. Endocrinol..

[16] Shibutani S.T., Saitoh T., Nowag H., Münz C., Yoshimori T. (2015). Autophagy and autophagy-related proteins in the immune system. Nat. Immunol..

[17] Liang S., Wu Y.S., Li D.Y., Tang J.X., Liu H.F. (2021). Autophagy in Viral Infection and Pathogenesis. Front. Cell Dev. Biol..

[18] Abdoli A., Alirezaei M., Mehrbod P., Forouzanfar F. (2018). Autophagy: The multi-purpose bridge in viral infections and host cells. Rev. Med. Virol..

[19] Shi J., Luo H. (2012). Interplay between the cellular autophagy machinery and positive-stranded RNA viruses. Acta Biochim. Biophys. Sin..

[20] Leonardi L., Sibéril S., Alifano M., Cremer I., Joubert P.E. (2021). Autophagy Modulation by Viral Infections Influences Tumor Development. Front. Oncol..

[21] Wild P., Farhan H., McEwan D.G., Wagner S., Rogov V.V., Brady N.R., Richter B., Korac J., Waidmann O., Choudhary C. (2011). Phosphorylation of the autophagy receptor optineurin restricts Salmonella growth. Science.

[22] Alirezaei M., Flynn C.T., Wood M.R., Whitton J.L. (2012). Pancreatic acinar cell-specific autophagy disruption reduces coxsackievirus replication and pathogenesis in vivo. Cell Host Microbe.

[23] Kemball C.C., Alirezaei M., Flynn C.T., Wood M.R., Harkins S., Kiosses W.B., Whitton J.L. (2010). Coxsackievirus infection induces autophagy-like vesicles and megaphagosomes in pancreatic acinar cells in vivo. J. Virol..

[24] Tabor-Godwin J.M., Tsueng G., Sayen M.R., Gottlieb R.A., Feuer R. (2012). The role of autophagy during coxsackievirus infection of neural progenitor and stem cells. Autophagy.

[25] Hansen T.E., Johansen T. (2011). Following autophagy step by step. BMC Biol..

[26] Galluzzi L., Baehrecke E.H., Ballabio A., Boya P., Bravo-San Pedro J.M., Cecconi F., Choi A.M., Chu C.T., Codogno P., Colombo M.I. (2017). Molecular definitions of autophagy and related processes. EMBO J..

[27] Gómez-Sánchez R., Pizarro-Estrella E., Yakhine-Diop S.M., Rodríguez-Arribas M., Bravo-San Pedro J.M., Fuentes J.M., González-Polo R.A. (2015). Routine Western blot to check autophagic flux: Cautions and recommendations. Anal. Biochem..

[28] Liang X.H., Kleeman L.K., Jiang H.H., Gordon G., Goldman J.E., Berry G., Herman B., Levine B. (1998). Protection against fatal Sindbis virus encephalitis by beclin, a novel Bcl-2-interacting protein. J. Virol..

[29] Liu Y., Zhou T., Hu J., Jin S., Wu J., Guan X., Wu Y., Cui J. (2022). Targeting Selective Autophagy as a Therapeutic Strategy for Viral Infectious Diseases. Front. Microbiol..

[30] Wong J., Zhang J., Si X., Gao G., Mao I., McManus B.M., Luo H. (2008). Autophagosome supports coxsackievirus B3 replication in host cells. J. Virol..

[31] Staring J., von Castelmur E., Blomen V.A., van den Hengel L.G., Brockmann M., Baggen J., Thibaut H.J., Nieuwenhuis J., Janssen H., van Kuppeveld F.J. (2017). PLA2G16 represents a switch between entry and clearance of Picornaviridae. Nature.

[32] Shi Y., He X., Zhu G., Tu H., Liu Z., Li W., Han S., Yin J., Peng B., Liu W. (2015). Coxsackievirus A16 elicits incomplete autophagy involving the mTOR and ERK pathways. PLoS ONE.

[33] Huang S.C., Chang C.L., Wang P.S., Tsai Y., Liu H.S. (2009). Enterovirus 71-induced autophagy detected in vitro and in vivo promotes viral replication. J. Med. Virol..

[34] Chang H., Li X., Cai Q., Li C., Tian L., Chen J., Xing X., Gan Y., Ouyang W., Yang Z. (2017). The PI3K/Akt/mTOR pathway is involved in CVB3-induced autophagy of HeLa cells. Int. J. Mol. Med..

[35] Luo X.N., Yao H.L., Song J., Song Q.Q., Shi B.T., Xia D., Han J. (2018). Coxsackievirus B3 Infection Triggers Autophagy through 3 Pathways of Endoplasmic Reticulum Stress. Biomed. Environ. Sci..

[36] Alirezaei M., Flynn C.T., Wood M.R., Harkins S., Whitton J.L. (2015). Coxsackievirus can exploit LC3 in both autophagy-dependent and -independent manners in vivo. Autophagy.

[37] Shi J., Wong J., Piesik P., Fung G., Zhang J., Jagdeo J., Li X., Jan E., Luo H. (2013). Cleavage of sequestosome 1/p62 by an enteroviral protease results in disrupted selective autophagy and impaired NFKB signaling. Autophagy.

[38] Ylä-Anttila P. (2021). Autophagy receptors as viral targets. Cell. Mol. Biol. Lett..

[39] Nalbandian A., Llewellyn K.J., Nguyen C., Yazdi P.G., Kimonis V.E. (2015). Rapamycin and chloroquine: The in vitro and in vivo effects of autophagy-modifying drugs show promising results in valosin containing protein multisystem proteinopathy. PLoS ONE.

[40] Jackson W.T., Giddings T.H., Taylor M.P., Mulinyawe S., Rabinovitch M., Kopito R.R., Kirkegaard K. (2005). Subversion of cellular autophagosomal machinery by RNA viruses. PLoS Biol..

[41] Sun P., Zhang S., Qin X., Chang X., Cui X., Li H., Zhang S., Gao H., Wang P., Zhang Z. (2018). Foot-and-mouth disease virus capsid protein VP2 activates the cellular EIF2S1-ATF4 pathway and induces autophagy via HSPB1. Autophagy.

[42] Ga Y.J., Go Y.Y., Yeh J.Y. (2024). Coding-complete genome sequence and phylogenetic analysis of Coxsackievirus B3 isolated from South Korea. Microbiol. Resour. Announc..

[43] Choi J., Chen J., Schreiber S.L., Clardy J. (1996). Structure of the FKBP12-rapamycin complex interacting with binding domain of human FRAP. Science.

[44] Hay N., Sonenberg N. (2004). Upstream and downstream of mTOR. Genes Dev..

